# Deciphering the functions of NELL2 in tumorigenesis and beyond

**DOI:** 10.3389/fimmu.2026.1780909

**Published:** 2026-04-01

**Authors:** Bingbing Li, Cong Fu, Yuning Ren, Xiaoling Zhang

**Affiliations:** 1 Key Laboratory of Organ Regeneration and Transplantation of Ministry of Education, First Hospital of Jilin University, Changchun, China; 2 National-Local Joint Engineering Laboratory of Animal Models for Human Disease, First Hospital of Jilin University, Changchun, China

**Keywords:** immunology, NELL2, oncogene, therapy, tumor suppressor

## Abstract

NELL2, a neuron-specific secreted glycoprotein, has emerged as a key regulator in multiple physiological and pathological contexts, including neurodegeneration, immunity, reproduction and cancer. Despite its diverse roles, the context-dependent duality of NELL2-acting as both an oncogene and tumor suppressor, a neuroprotectant and contributor to hyperexcitability-remains mechanistically unclear. Moreover, the tissue-specific signaling cross-talk of NELL2 complicates its therapeutic targeting. Research on NELL2 holds transformative potential for precision medicine, offering insights into cancer therapy, neurodegenerative disease intervention, and infertility treatment, while highlighting the need for mechanistic clarity and context-specific strategies to realize its diagnostic and therapeutic promise. Here, we consolidated research advances of NELL2, delineating its structural features, regulatory networks, and context-dependent roles in neurodegeneration, immunity, reproduction and cancer. By highlighting unresolved mechanistic ambiguities and therapeutic translatability, our work helps to illuminate potential of NELL2 as a diagnostic marker and therapeutic target across diverse diseases.

## 1 Introduction

Neural epidermal growth factor-like like 2 (NELL2), a neuron-specific secreted glycoprotein of the NEL family, is characterized by conserved structural domains (1) which enable NELL2 to mediate critical interactions with signaling molecules such as protein kinase C (PKC) and receptors like Robo3, positioning it as a pivotal regulator in neurodevelopment, synaptic plasticity, and extracellular matrix dynamics (2–4). NELL2 exhibits widespread expression during embryonic neurogenesis, particularly in the hippocampus, spinal cord, and sensory ganglia, while its expression declines postnatally but persists in specific regions (5, 6). Structurally, NELL2 shares homology with thrombospondins, suggesting an evolutionary conservation of roles in neurodevelopment (1). The neuron-specific localization and dynamic expression patterns of NELL2 underscore its importance in processes ranging from axon guidance to synaptic homeostasis (7).

The regulation of NELL2 is multifaceted, involving transcriptional, epigenetic, and post-transcriptional mechanisms. At the transcriptional level, NELL2 is directly activated by E2F transcription factor 1 (E2F1) in cancers such as non-small cell lung cancer (NSCLC), while its expression can be suppressed by promoter hypermethylation in renal cell carcinoma (RCC) (8–10). Epigenetic modifiers like enhancer of Zeste Homolog 2 (EZH2) further modulate NELL2-mediated pathways, influencing its oncogenic or tumor-suppressive outcomes (11). Post-transcriptionally, NELL2 is targeted by non-coding RNAs, such as miR-22 and tRNA-derived fragments (e.g., tRF-3017A), which fine-tune its translation in a cell context-dependent manner (12, 13). Additionally, splice variants like the cytosolic cNELL2 exhibit distinct regulatory roles by inhibiting PKCβ1 activity in astrocytes, highlighting the complexity of NELL2 regulation across tissues and disease states (14).

Functionally, NELL2 demonstrates remarkable duality across physiological and pathological contexts. It was found that NELL2 acts as an oncogene in NSCLC and Ewing sarcoma by driving proliferation via E2F1 or stabilizing chromatin remodeling complexes, yet functions as a tumor suppressor in hepatocellular carcinoma (HCC) through other pathways like Notch inhibition (8, 9, 11, 15). Beyond cancer, NELL2 modulates neuroendocrine functions, including gonadotropin-releasing hormone (GnRH)-dependent puberty initiation, and contributes to neurodegenerative diseases such as Alzheimer’s disease (AD) by exacerbating neuronal hyperexcitability (16, 17). In immunity, NELL2 promotes Th2-skewed inflammation in atopic dermatitis (ADs) and psoriasis (18), while in reproduction, it governs lumicrine signaling essential for sperm maturation (19, 20). Such functional versatility is further shaped by tissue-specific signaling cross-talk and microenvironmental cues.

Here, we thoroughly summarized and discussed the recent advances in the structural features, regulation of NELL2 and its context-dependent roles in cancer, neurodegeneration, immunity, and reproduction. Therefore, our work facilitates to illuminate the potential of NELL2 as a diagnostic marker and therapeutic target across diverse diseases through highlighting unresolved mechanistic ambiguities and therapeutic translatability of NELL2.

## 2 Structure and expression features of NELL2

NELL2 is the mammalian homologue of chicken NEL (neuro epidermal growth factor-like molecule) and belongs to the NEL family which was originally isolated from a chicken cDNA library and named for its EGF-like structural domain and high expression in neural tissues. Subsequently, two related genes were identified in mammals, named NELL (NEL-like) 1 and 2 (3). The cDNA of NELL2 is 3198bp and encodes a secretory glycoprotein of 816 amino acids, which shares 80% amino acid homology with the chicken NEL gene, and 55% similarity with NELL1 (2, 3).

### 2.1 The structure domains of NELL2

**Figure 1:**
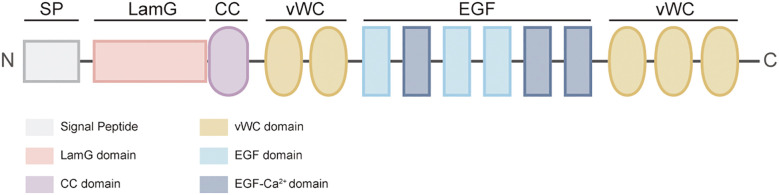
The structure of NELL2. NELL2 consists of five basic structures which include the Lam G and CC structural domains (TSP-N domain), and the second, fifth, and sixth EGF domains, which contain calcium-binding sites and are the main active regions.

**Figure 2:**
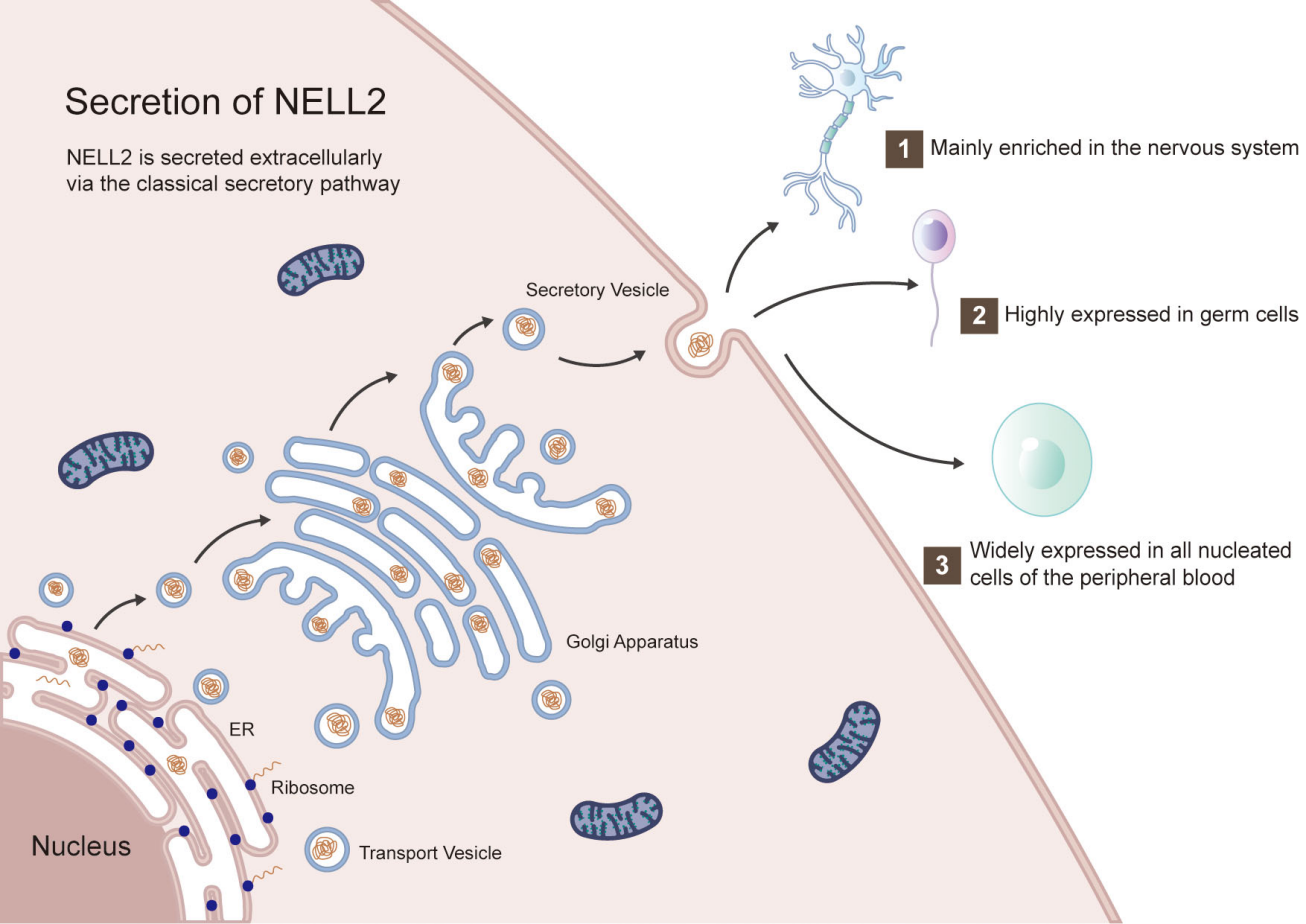
The secretory process of NELL2. Post-transcriptionally modified NELL2 is localized in the ER and Golgi, and ultimately is transported by secretory vesicles to the extracellular compartment, where they perform various physiological functions in different cells.

NELL2 is a neuron-specific secreted glycoprotein characterized by an N-terminal thrombospondin-1 (TSP-1)-like module (homologous to TSP-1) that possesses heparin-binding activity, thereby affecting its interaction with cell surface proteoglycans. It contains a coiled-coil (CC) domain that mediates homotrimer formation, followed by five von Willebrand factor type C (vWC) domains and six conserved EGF-like domains (designated EGF 1~6). These EGF domains each contain six conserved cysteine residues, with the second, fifth, and sixth EGF domains featuring Ca^2+^-binding sites. The key binding regions located within the EGF2 and EGF3 domains, which are also the core regions mediating interactions with PKC (especially the 2nd, 5th and 6th) (1, 2, 4, 21) (**Figure 1**). NELL2 is secreted via the classical secretory pathway and is mainly localized in the endoplasmic reticulum (ER), Golgi apparatus and secretory vesicles, with partial secretion into the extracellular compartment, and the vesicles are transported by microtubule-dependent bidirectional motility, not actin (14, 22). The N-terminal 29 amino acids are critical for NELL2 secretion (**Figure 2**). Notably, the secreted NELL2 may regulate neuronal differentiation and survival in a paracrine-autocrine manner, and the interaction of NELL2 with the microtubule-actin cross-linking factor 1 (Macf1) may be involved in regulating its trafficking (22, 23).

### 2.2 The expression profile of NELL2

NELL2 is widely expressed in all nucleated cells in normal peripheral blood, including B cells, T cells, monocytes and natural killer cells, at levels comparable to those in brain tissue. In contrast, the expression of *NELL2* was not detected in early B cell precursors (e.g., stem cells, B cell progenitors, and pre-B cells) in the bone marrow, suggesting that its expression is tightly regulated by developmental stage and cell lineage (24). In the immune system, NELL2 is mainly expressed in T-cell clusters, and its relative expression gradually increases during T-cell differentiation, suggesting that it plays a key role in T-cell differentiation (25). Importantly, NELL2 is positively correlated with immune cells, such as M0/M1 macrophages and CD4^+^ memory-activated T cells, and negatively correlated with regulatory T cells (Tregs) and activated natural killer (NK) cells, suggesting that it may be involved in regulating immune cell infiltration and function (26).

Moreover, NELL2 is involved in the neuronal differentiation and muscle lineage specialization, and is highly expressed in specific regions of the central nervous system (CNS) such as the spinal cord, retina, midbrain and hindbrain, and is enriched in the differentiated neuronal layer (mantle layer) but not in the proliferating progenitor zone (ventricular layer). Furthermore, NELL2 was found to be highly expressed in the immature dorsal root ganglion (DRG) and cranial sensory ganglia (e.g., trigeminal ganglia), and specifically labels hypaxial muscle progenitors at the lateral margins of somatic ganglia (which migrate to the limb and body wall to form skeletal muscle), whereas epaxial muscle progenitors (which form paraspinal muscle) do not express NELL2, reflecting its multiple regulatory roles in developmental biology (27).

Notably, two NELL2 genes (NELL2a and NELL2b) encoding multifunctional glycoproteins with EGF-like structural domains are present in rainbow trout. It was found that the NELL2a is a key gene for rainbow trout in response to environmental and biological stresses. Phylogenetic analyses indicated that the NELL gene may exist in two forms in scleractinian fish: NELL1/NELL2 (directly homologous to mammals, e.g., pufferfish, spiny dogfish); and NELL2a/NELL2b (fish-specific gene duplication products, e.g., zebrafish, salmonids) (28).

## 3 The regulation of NELL2

NELL2 is involved in neurodevelopmental and potentially oncogenic processes by binding to the NH_2_-terminal variable region of PKC isoforms (β1, ϵ, ζ), and its function depends on EGF-like structural domain-mediated protein interactions and phosphorylation of PKC (1, 2). Interactions with PKC may affect neuronal function by modulating correlated signaling pathways (e.g., Ca^2+^-dependent signaling), and PKC-mediated phosphorylation may promote carcinogenesis by activating downstream signaling pathways (e.g., cell proliferation or anti-apoptotic pathways) (2).

E2F1 transcription factors promote *NELL2* expression by directly binding to the promoter region of NELL2 and activating its transcription. Notably, the m6A demethylase fat mass and obesity-associated protein (FTO) stabilizes and upregulates the expression of *E2F1* by inhibiting the m6A modification of E2F1 mRNA, which in turn enhances the transcriptional activity of NELL2 to form the pro-cancer FTO/E2F1/NELL2 axis. Importantly, the lncRNA-HIT increases the binding efficiency of E2F1 in the NELL2 promoter region by interacting with E2F1 protein, which further enhances the transcriptional activity of NELL2 (8, 9).

Interestingly, the mouse NELL2 promoter contains two half-E2 response elements (half-EREs, located at sites -223 and -1047) and several SP1 response elements (SP1REs), and the E2 forms a complex with ERα/ERβ (dependent on the half-EREs and part of the SP1REs (e.g. sites -209 and -430)), through which the half-EREs directly activate NELL2 transcription (29). Moreover, NELL2 is a key regulator of retinoic acid (RA)-induced neural differentiation, and RA binds to the two half-RA response elements (RARE) on the NELL2 promoter, significantly increasing the mRNA and protein levels of NELL2 (30).

**Figure 3:**
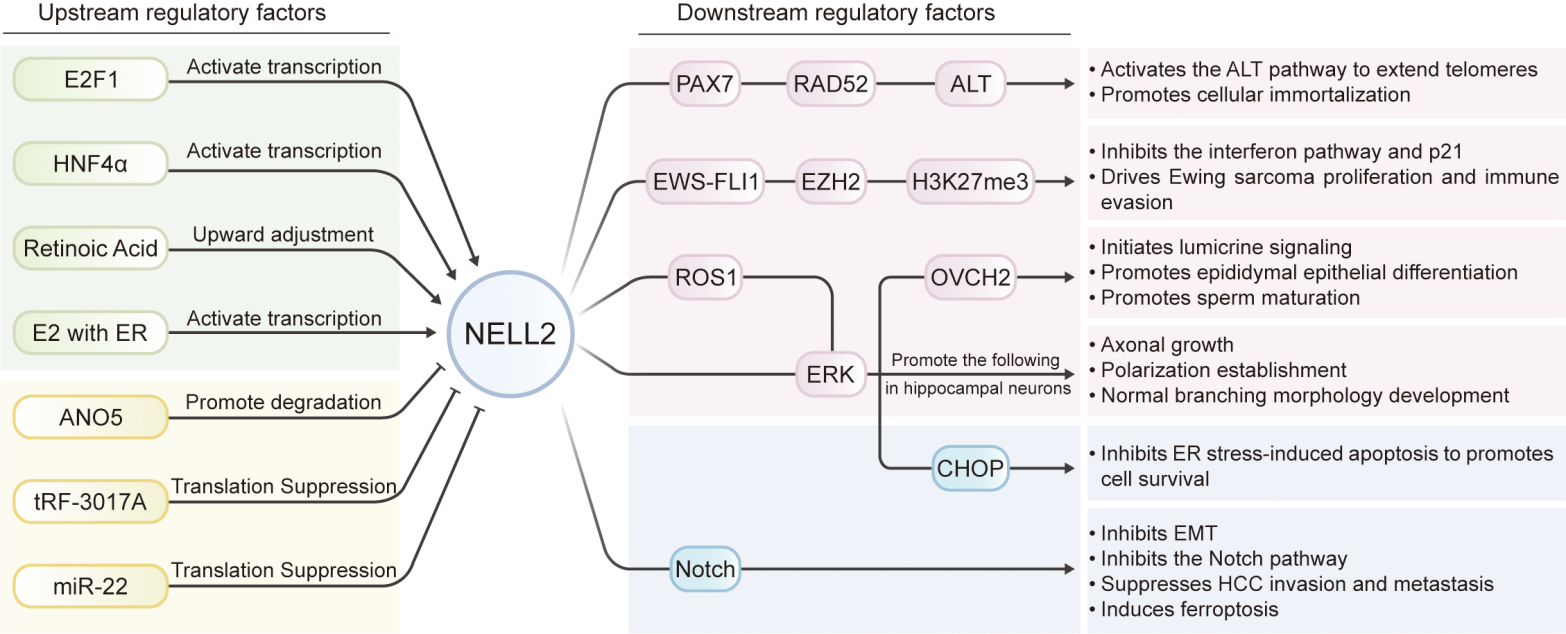
The upstream regulation factors of NELL2 and the downstream factors regulated by NELL2. The transcription of NELL2 is activated by factors including E2F1, HNF4α, retinoic acid and estrogen receptor complexes (E2 with ERα/ERβ), while its expression or stability is negatively regulated by ANO5, IRF-3017A and miR-22. NELL2 functions by extending telomeres via activation of the ALT pathway, suppressing interferon signaling and p21 expression, initiating sperm maturation via the lumicrin signaling pathway, promoting hippocampal neuronal axon growth and morphogenesis, inhibiting endoplasmic reticulum stress-induced apoptosis, and blocking cancer metastasis while inducing ferroptosis by inhibiting the Notch pathway.

Notably, NELL2 has been identified as a direct target of microRNA-22 (miR-22). miR-22 is a highly conserved non-coding small RNA molecule that binds to the 3’ untranslated region (3’UTR) of NELL2 mRNA through its seed sequence, leading to degradation or translational repression of NELL2 mRNA, a process that is reversed by the activated vitamin D/vitamin D receptor (VDR) signaling pathway (12). Notably, the tRF-3017A, a small RNA fragment (19 nt) derived from the 3’ end of tRNA-Val-TAC, could bind to Argonaute (AGO) protein to form an RNA-induced silencing complex (RISC) that directly targets the 3’UTR of NELL2 mRNA, resulting in translational repression or degradation of NELL2 mRNA, and subsequent downregulation of NELL2 protein expression (13). Furthermore, hypermethylation of CpG islands in the promoter region of NELL2 also leads to its transcriptional silencing (10) (**Figure 3**).

## 4 Physiological functions of NELL2

### 4.1 NELL2 in neurodevelopment

NELL2 is a brain-specific gene that is mainly expressed in neurons and their progenitors and is closely associated with neural differentiation and neurogenic tumors. The NELL2 gene is localized to the region of chromosome 12q13.11-q13.12, adjacent to the region of known extracellular matrix-associated genes, and it is hypothesized that NELL2 may be involved in neuronal signaling. And the similarity of NELL2 structural features to the chicken NEL gene suggests that the two may be evolutionarily conserved and involved in neurodevelopmental regulation. NELL2 is highly expressed in human fetal and adult brain tissue, and weakly expressed in fetal kidney, with no signal detected in other tissues. NELL2 mRNA showed a major band of 3.6 kb, but a small unknown fragment of 0.8 kb was also detected in fetal liver (3). The widespread expression of *NELL2* in the embryonic nervous system suggests that it is involved in the processes of neural plate formation, neuronal differentiation and axon guidance, similar to the neurodevelopmental function of TSP-1. In adulthood, it is specifically highly expressed only in adult brain regions. This suggests that NELL2 may be involved in neuroplasticity, neural development and differentiation, modulation of motor function, and maintenance of synaptic homeostasis (5). NELL2 ensures the accuracy of midline crossing by repelling axons in ventral regions of the spinal cord and guiding spinal cord coaxons to precisely bypass areas with higher expression of *NELL2* (4). Moreover, the association of NELL2 with calcium signaling and synaptic vesicle release suggests a possible role in neurodegenerative diseases or synaptic dysfunction (5).

### 4.2 NELL2 in embryonic development

In rats, NELL2 mRNA is detected as early as embryonic development and gradually increases during embryonic development, and reaches peak at E20. Given that NELL2 was mainly distributed in the CNS, including the ventricular zone and differentiation zone of the spinal cord, medulla oblongata and pons, suggesting its involvement in neurogenesis and neuronal differentiation. The expression of *NELL2* gradually decreases after birth, but remains high in certain brain regions in adult rats, including the olfactory bulb, hippocampus (regions CA1 and CA3), dentate gyrus, cerebellar cortex, nucleus accumbens and inferior olivary nucleus, etc. Notably, NELL2 is expressed exclusively in neurons and is not seen in glial cells or white matter regions (5). The NELL2 protein is specifically expressed in the rat brain, and the non-glycosylated and glycosylated forms correspond to sizes of 90 kDa and 140 kDa, respectively. It is most abundantly expressed in the hippocampus and cerebral cortex, moderately in the olfactory bulb and hypothalamus, and to a lesser extent in the thalamus, cerebellum, and spinal cord, with different proportions of the two protein bands in different brain regions (e.g, the olfactory bulb predominantly at 90 kDa and the hypothalamus predominantly at 140 kDa) (1). Outside the nervous system, NELL2 is not expressed in normal peripheral blood mononuclear cells, but its expression has been detected in peripheral blood samples containing CD34^+^ hematopoietic stem cells (31).

Interestingly, the cNELL2 is a non-secretory splice variant of NELL2 that lacks the signal peptide sequence by selective splicing with deletion of exon 3 (containing the signal peptide terminus), and cannot be secreted extracellularly and is retained in the cytoplasm (14). The mRNA and protein size between the two splice variants showed a small difference, with only 129 bp missing in cNELL2 (32). It was found that the cNELL2 acts as an endogenous inhibitor of PKCβ1 by binding to its N-terminal pseudo-substrate (PS) domain to inhibit the membrane translocation of PKCβ1 (induced by Ca²^+^/UTP), thereby blocking its downstream ERK phosphorylation and other signaling pathways (14).

Notably, the NELL2-TSP is a C-terminal truncated splice variant of NELL2 that is produced by variable shearing, is specifically expressed in the brain, and may be involved in the dynamic balance of neurodevelopment as a negative regulator by binding to and inhibiting the secretion and function of wild-type NELL2 (33). However, the NELL2-TSP loss the secretion and function of wild-type NELL2 due to the absence of EGF-like structural domain. Importantly, the structural domain of TSP-N is also the key functional domain of both NELL2 and NELL2-TSP that mediates the binding of NELL2-TSP to wild-type NELL2 to interfere with its secretory or functional structural domains.

### 4.3 NELL2 in reproduction regulation

NELL2, a core ligand of the lumicrine signaling pathway, can be secreted from testicular germ cells, and the secreted NELL2 transports to the epididymis by the luminal fluid stream of the vas deferens, and forms a tight heterodimeric complex with NELL2-interacting cofactor for lumicrine signaling (NICOL). Subsequently, the heterodimeric complex binds to the cell surface receptor tyrosine kinase ROS1 on the epididymal initial segment (IS), and triggers downstream signals (e.g., ERK phosphorylation) to induce the expression of genes related to epididymal epithelial differentiation and spermatid maturation (e.g., OVCH2, ADAM28) (19, 34–38). Subsequently, the epididymal sperm surface protein ADAM3 (which critically regulates sperm migration to the oviducts) is processed from the precursor (100 kDa) to the mature form (30 kDa), allowing sperm to acquire the ability to migrate and fertilize. NELL2 is prominently expressed in germ cells (e.g., spermatocytes, spermatozoa) of the human testis, especially in late germ cells (e.g., rough-lineage spermatocytes) located on the medial side of the blood-testis barrier (BTB), fulfilling the secretory localization of a lumicrine signaling ligand (19). Genes regulated by NELL2 mainly belong to the gene family (e.g., β-defensins, lipocalins) that emerged after the evolutionary stage of Amniota and are essential for epididymis-specific functions. Moreover, the NELL2-OVCH2 signaling pathway is highly conserved in mammals, and despite differences in the structure of the human epididymis, the molecular mechanisms are highly consistent, and its absence results in abnormal epididymal function across species (19, 34).

Moreover, NELL2 is a key molecule in the E2-regulated development of sexually dimorphic nucleus of the preoptic area (SDN-POA) neurons, and its normal expression during the neonatal period is essential for the formation of sexually dimorphic structures in the brain of male rats and the adult sexual behavior. The volume of the SDN-POA in male rats with NELL2 blockade was significantly reduced. The frequency of mounting and intromission behaviors was reduced in adult male rats, but sexual preference (attraction to females) was unaffected (39).

NELL2 also plays critical roles in the regulation of the estrous cycle in female rats. E2 directly regulates the transcription of *NELL2* through E2 receptor alpha (ERα), and NELL2 acts as a downstream effector molecule of E2 signaling in ERα-positive neurons, meanwhile, is specifically expressed in glutamatergic neurons (non-GnRH neurons), through a serum E2-dependent mechanism, affecting kisspeptin 1 neuronal activity by regulating glutamate release to maintain normal activity of GnRH neurons (40). Notably, NELL2 is differentially expressed in the hypothalamus after E2 treatment in the neonatal period, whose expression is regulated by E2 and has a prepubertal peak, may play a role in pubertal initiation and hypothalamic sexual differentiation through the PKC-Ca²^+^ signaling pathway (41).

**Figure 4:**
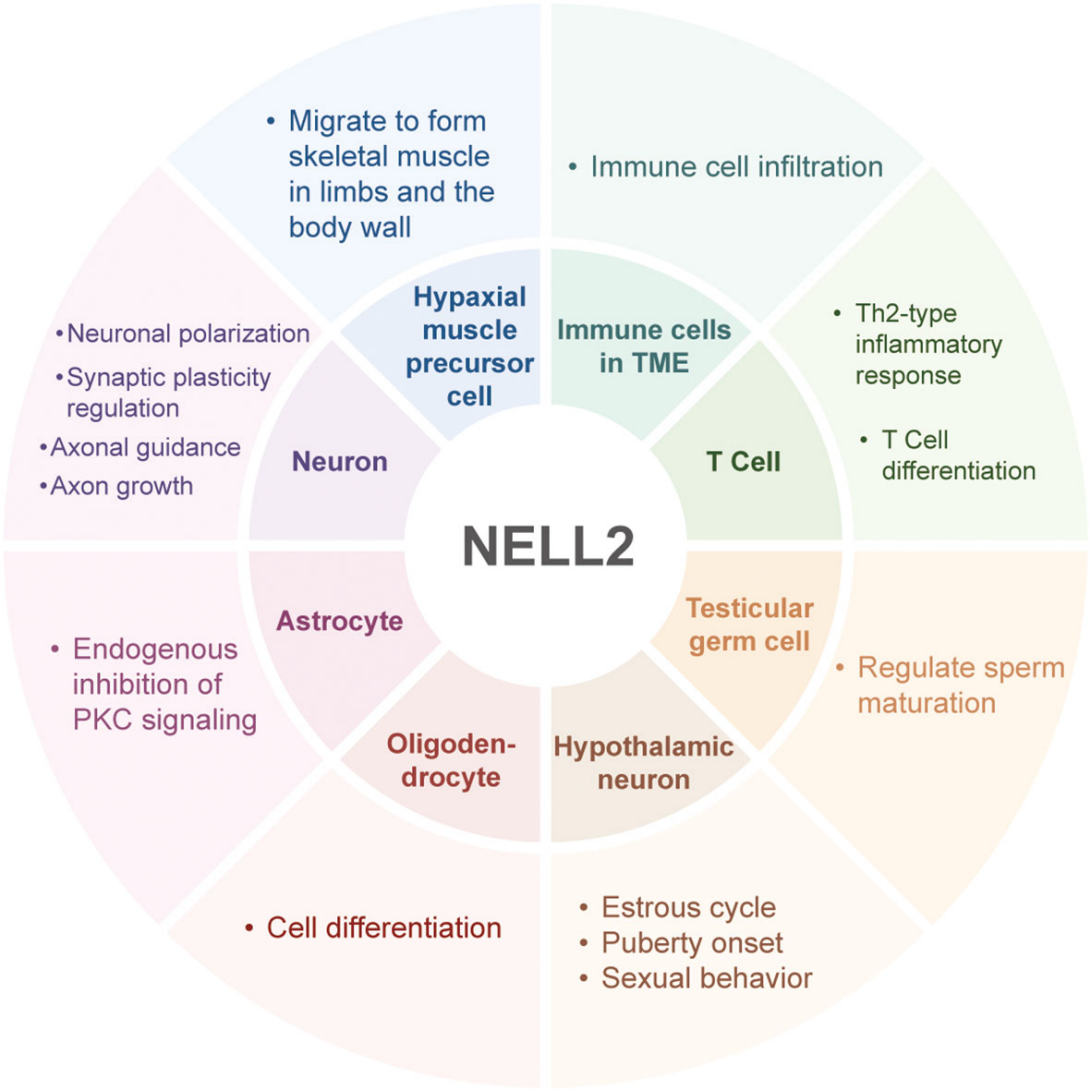
The physiological roles of NELL2. NELL2 plays multiple roles in the nervous, immune, and reproductive systems of mammals, holding significant importance for human development from embryonic stages through adolescence, as well as for immune system function and disease regulation.

NELL2 indirectly regulates the maturation process of fish oocytes by affecting the release of GnRH (42). Moreover, NELL2 is located on chromosome 1 in chickens, and its neighboring SNPs are significantly associated with egg production. Therefore, NELL2 affects the reproductive cycle by regulating GnRH synthesis and secretion (43, 44). White striping (WS) is a common myopathy in broiler breast muscle, NELL2 is located in a quantitative trait locusregion associated with body weight, pectoral muscle weight, intramuscular fat content, and muscle fiber characteristics (e.g., diameter and density), suggesting that it may influence the development of WS through regulation of muscle growth and metabolism (45) (**Figure 4**).

### 4.4 NELL2-mediated downstream signaling pathways

NELL2 regulates the normal development and morphology of hippocampal neuronal axons through the ERK signaling pathway, and its loss of function leads to abnormalities that may impair neural information transmission (46). Moreover, NELL2 induces axon growth and neuronal polarization through activation of the ERK signaling pathway and has specific regulatory effect on axon development in a dose-dependent manner (18, 47). Overexpression of *NELL2* accelerated the progression of neurons from the polyneuronal stage (stage 2) to the axonal differentiation stage (stage 3). The increased axon length (average increase of about 50%), and the decreased number of synapses of individual neurons reflect its roles in facilitating the establishment of neuronal polarity (46) (**Figure 3**).

#### 4.4.1 Proenkephalinogen

The proenkephalinogen (PPE) is a precursor protein of enkephalins (ENKs) which include Leu-ENK and Met-ENK, play a role in regulating various physiology and are especially widely expressed in the brain. NELL2 regulates the expression of PPE in a dual manner through its EGF-like structural domain and spatial localization. ER-localized NELL2 down-regulates PPE transcription by inhibiting the Ca²^+^-PKC-ERK-cFos pathway, hence, overexpression of *NELL2* causes PPE transcriptional repression. However, the secreted NELL2 promotes PPE transcription by activating the ERK pathway. Therefore, the dual regulation of PPE by NELL2 suggests that NELL2 plays a complex role in regulating neuropeptide dynamics (48).

#### 4.4.2 Roundabout

The Roundabout (Robo) family is a conserved class of transmembrane receptor proteins primarily involved in the regulation of axon guidance and angiogenesis in the nervous system. NELL2, as a repulsive ligand for Robo3, acts synergistically with Slit inhibition and Netrin enhancement to ensure that spinal axons are precisely guided to the midline by antagonizing Robo1/2. Robo3 controls axon guidance through three mechanisms: 1) mediating NELL2 repulsion to prevent axons from invading NELL2-overexpressing regions; 2) inhibiting Slit repulsion by antagonizing Robo1/2 signaling; and 3) enhancing Netrin attraction by promoting deleted in colorectal cancer (DCC) signaling. This redundant mechanism ensures that axons cross the midline with high fidelity (7). NELL2 binds specifically to the first fibronectin type III (FN) structural domain of Robo3 (FN1) via the EGF2 and EGF3 structural domains (4, 49). The affinity of NELL2 for Robo3 has a direct impact on axonal repulsion. The trimerization of NELL2 formed through the CC structural domain is a key mechanism for signal amplification, with oligomerization significantly enhancing repulsive signals. The pH-dependent binding of NELL2 to ROBO3 is achieved by protonation of Glu592, which may be modulated by pathological or specific physiological conditions (e.g., inflammation, tumor microenvironment). Therefore, the intracerebral pH fluctuations may affect neurodevelopment or disease (e.g., epilepsy, psychiatric disorders) by modulating NELL2-ROBO3 signaling (49, 50).

#### 4.4.3 Microtubule-actin cross-linking factor 1

NELL2 ensures the precise formation of binocular visual projections by repelling contralateral retinal ganglion cell (RGC) axons and facilitating the competitive occupancy of ipsilateral axons (51). It was found that only the secreted NELL2 isoform (140 kDa glycosylated form and 90 kDa non-glycosylated form) was present in the retina, and bound directly to Macf1, which was highly expressed in RGCs and their dendrites. These findings suggested that Macf1 may be involved in the function of NELL2 by modulating cytoskeletal dynamics or the Wnt signaling pathway (23).

#### 4.4.4 long-term potentiation

NELL2 was reported to negatively regulate the synaptic plasticity by inhibiting long-term potentiation (LTP) in the dentate gyrus of the hippocampus, playing an important role in maintaining the homeostasis of synaptic plasticity. Hence, NELL2 is a key regulator of hippocampus-dependent spatial learning, and the deficiency of *NELL2* results in reduced learning capability due to the increased LTP (52). Notably, previous studies have shown that NELL2 promotes neuronal survival by regulating the ERK/JNK signaling pathway, which is involved in the regulation of LTP. In line with this finding, *NELL2* deletion may reduce JNK activity and increase ERK activity, which enhances LTP and thus disrupts the integration of spatial information in the hippocampus (52, 53).

NELL2 exhibits a remarkable regional and neuronal phenotype-specific expression pattern in the adult rat brain and may play a key role in regulating neuroplasticity and mature neuronal function. The differential expression of *NELL2* in the cerebellum and hippocampus suggests that NELL2 may be involved in the regulation of neuronal plasticity through a calcium-dependent mechanism, particularly in the maintenance of mature neuronal function (54).

#### 4.4.5 PKCβ1

It was found that the *NELL2* expression is upregulated in oligodendrocytes of the human fetal brain and spinal cord, and its expression increases significantly with oligodendrocytes maturation, positively correlating with developmental stage. Moreover, *NELL2* is expressed in human induced pluripotent stem cell (hiPSC)-derived brain-like organoids, particularly in neural stem cells, neurons, and oligodendrocytes. Importantly, NELL2 may exert it roles by regulating the PKCβ1 signaling pathway, which is known to be a key factor affecting oligodendrocyte differentiation (55).

## 5 The roles of NELL2 in tumors

### 5.1 The roles of NELL2 as an oncogene

#### 5.1.1 E2F1

NELL2 was identified as one of the target genes of E2F1 transcription factor, which is a key component of the lncRNA-HIT/E2F1 and FTO/E2F1 regulatory axis. E2F1 plays critical roles in tumorigenesis and was found to regulate the proliferation of non-small cell lung cancer cells (8, 9). In NSCLC cells, E2F1 can directly bind to the promoter region of NELL2 gene, which is dependent on the lncRNA-HIT-mediated E2F1 recruitment (8). Meanwhile, the FTO-mediated demethylation of E2F1 m6A drives *NELL2* expression which promotes the proliferation, migration and metastasis of NSCLC cells (9). Therefore, targeting lncRNA-HIT/E2F1/NELL2 or the FTO/E2F1/NELL2 axis might be a novel therapeutic strategy in the treatment of NSCLC.

#### 5.1.2 EWS-FLI1

NELL2 could transcriptionally activate the transcription factor EWS-FLI1 through autocrine signaling to promote tumor proliferation (11, 56). In details, NELL2 stabilizes the BAF chromatin remodeling complex by inactivating Cdc42 to enhance EWS-FLI1 activity, which subsequently drives cell proliferation. NELL2 could also form a positive feedback loop with CD133 and EWS-FLI1 to maintain the highly proliferative phenotype. In cells, overexpression of *CD133* restored NELL2 expression and suppressed interferon genes. Moreover, inhibition of NELL2 induces cell growth arrest and concomitant activation of interferons, accompanied by significant upregulation of interferon β1, interferon-stimulated genes (Mx1, OAS1), and the cell cycle inhibitor p21 (CDKN1A).

#### 5.1.3 EZH2

NELL2 signal pathway mediates H3K27me3 modification through the downstream target histone methyltransferase, EZH2, and epigenetically silences endogenous retroviruses (ERVs) and the LINE-1 reverse transcription transposon (12). Silence of NELL2 reduces H3K27me3, which in turn derepresses viral components, leading to release of viral RNA and activation of interferon β1, interferon-stimulated genes (e.g., Mx1, OAS1) and the cell cycle inhibitory factor p21, leading to an antiviral response and growth arrest. Notably, inhibition of NELL2-EZH2 may provide a novel target for immunotherapy by activating viral mimicry and converting “cold tumors” to an immunosensitive state in Ewing sarcoma. The NELL2-EZH2 axis showed well response to therapy in a variety of cancers, but is insensitive in non-transformed cells (e.g. fibroblasts, endothelial cells) (11).

#### 5.1.4 Alternative lengthening of telomeres

**Figure 5:**
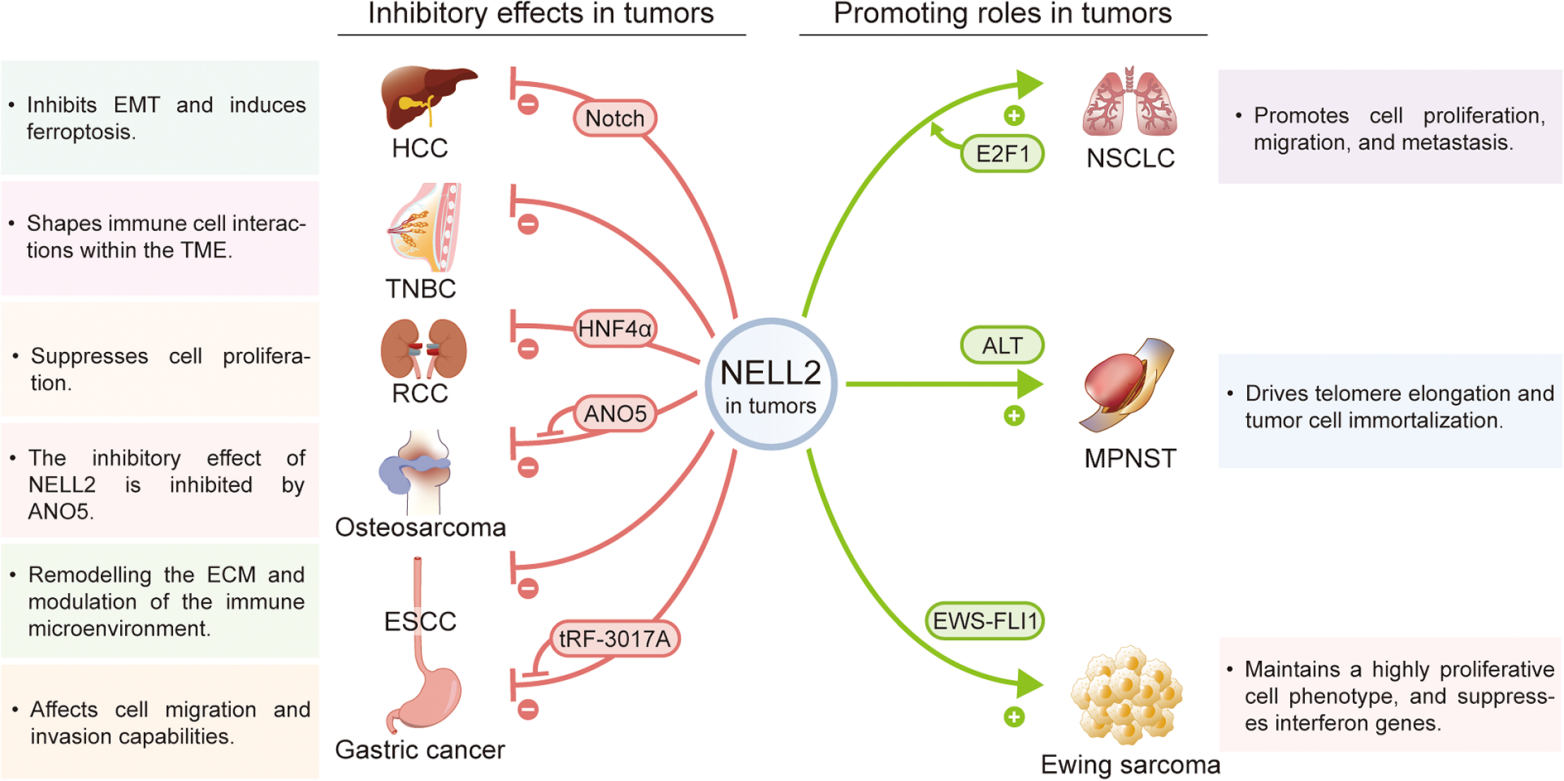
The dual roles of NELL2 in tumorigenesis. NELL2 generally functions as a tumor suppressor by inhibiting EMT via the Notch pathway, inducing ferroptosis, remodeling the ECM, and modulating the tumor TME. NELL2 inhibits the proliferation of RCC and gastric cancer. However, in specific contexts such as NSCLC, MPNST, and Ewing sarcoma, NELL2 promotes tumor progression by driving proliferation, migration, metastasis, and immune evasion through mechanisms including ALT pathway-mediated telomere elongation and suppression of interferon genes. The tumor-suppressive effect of NELL2 in osteosarcoma is inhibited by ANO5.

NELL2 could immortalize tumor cells through the alternative lengthening of telomeres (ALT) pathway. Mechanistically, NELL2 drives the malignant transformation of malignant peripheral nerve sheath tumors (MPNSTs) by transcriptionally activating PAX7, which in turn initiates the RAD52-dependent ALT pathway, leading to telomere lengthening and tumor cell immortalization, promoting tumor progression and poor prognosis (57). Therefore, the NELL2/PAX7/RAD52-ALT cascade reveals the critical role of NELL2 in the telomere maintenance in neurogenic tumors and provides a novel potential for targeted therapy (**Figure 5**).

### 5.2 The roles of NELL2 as tumor suppressor

In hepatocellular carcinoma (HCC), NELL2 exerts tumor suppressive function through inhibiting epithelial-mesenchymal transition (EMT) and inducing iron death via the Notch signaling pathway (15). Moreover, NELL2 is one of the key genes in the transcriptional regulatory network of HNF4α, which is involved in cell adhesion or cytoskeleton regulation through mediating the inhibitory effect of HNF4α on renal cell proliferation (58). Notably, tRF-3017A was highly expressed in gastric cancer tissues and cell lines. NELL2 is a key downstream target and negatively correlated with tRF-3017A, which in turn affects the migration and invasive ability of gastric cancer cells (13). Interestingly, ANO5 could promote tumor progression by directly binding to and promoting the degradation of NELL2. Therefore, restoring NELL2 expression or targeting ANO5 may be a new strategy for the treatment of osteosarcoma (59).

Notably, NELL2 might exert an inhibitory effect on tumor progression by modulating the immune microenvironment (26, 60). In esophageal squamous cell carcinoma (ESCC), NELL2 exhibits important diagnostic value and potential therapeutic relevance by participating in extracellular matrix (ECM) remodeling and immune microenvironment regulation. As a tumor-infiltrating lymphocyte (TIL)-associated key gene, *NELL2* expression was negatively correlated with tumor purity and negatively correlated with infiltration of immunosuppressive cells (such as M2-type macrophages and regulatory T cells) in ESCC, which may regulate immune cell infiltration in the tumor microenvironment (TME) (26). In triple-negative breast cancer (TNBC), NELL2 may be involved in immune escape or tumor progression by influencing immune cell interactions in the TME, which is closely associated with the prognosis of TNBC patients (60). Moreover, NELL2 has been identified as a hub gene of the brown module, which is associated with adaptive immunity involving activation of signaling pathways (e.g., TCR/BCR pathway) in T and B cells (61) (**Figure 5**).

## 6 The roles of NELL2 in non-tumor human diseases

### 6.1 Alzheimer’s disease

NELL2 plays a central role in the early pathology of apoE4-driven Alzheimer’s disease (AD). The overexpression of *NELL2* was found to promote hippocampal network hyperexcitability by inducing neuronal atrophy and electrophysiological abnormalities (17). NELL2 inhibition ameliorates the early pathological phenotype of AD which suggest that targeting NELL2 may offer a novel strategy to delay neurodegenerative lesions in apoE4-associated AD. Moreover, the expression of *NELL2* and its circRNA are upregulated in AD mouse models (e.g., 5XFAD) and in the cortex of AD patients. Furthermore, co-expression of *NELL2* and *MDGA2*, another AD-related gene, may synergistically contribute to the pathological phenotype (17).

### 6.2 Skin disease

Notably, NELL2 expression was significantly upregulated in T cell-rich psoriasis models and promoted keratinocyte proliferation through activation of the ERK1/2 signaling pathway. The inverse regulation of NELL2 by PTPRM promotes epidermal proliferation and formation of an inflammatory microenvironment in psoriasis (62). Moreover, NELL2 is a key marker of nerve regeneration common to chronic nodular pruritic dermatitis (CNPG) and ADs, and *NELL2* overexpression might be the underlying repair mechanism of chronic scratch-induced nerve damage and is closely associated with inflammatory pathways (e.g., IL-4/NF-κB). Therefore, the finding provides a molecular basis for distinguishing chronic pruritic dermatoses (i.e., CNPG and ADs) from neuropathic pruritus (i.e., BRP). In pruritic dermatoses, higher expression of NELL2 may be associated with nerve fiber damage and repair due to prolonged scratching, whereas no upregulation of NELL2 was observed in BRP because the neuropathic mechanisms do not depend on such regenerative responses (63).

### 6.3 Tuberculous meningitis

NELL2 is a key biomarker for the prognosis of patients with tuberculous meningitis (TBM), especially in the HIV-negative population, and may exacerbate disease progression by suppressing adaptive immune responses. The low expression of NELL2 is strongly correlated with an increased risk of death. NELL2 is abundantly expressed in brain tissues (e.g., hippocampus and cerebral cortex), lower levels of NELL2 in the cerebrospinal fluid of patients with TBM than in normal populations may reflect TBM-associated neuronal damage, with an increase after anti-tuberculosis treatment suggesting restoration of neurological function (61, 64).

### 6.4 Pulmonary fibrosis

NELL2 has been identified as a potential key gene in pulmonary fibrosis in COVID-19 patients. The expression of *NELL2* is significantly associated with the activation of pro-inflammatory cells, such as MDSCs and macrophages. Therefore, NELL2 may be involved in the pathological process of pulmonary fibrosis by modulating inflammatory responses (65).

## 7 Conclusion and discussions

The role of NELL2 in pathophysiology remains highly controversial, primarily due to its dual functionality in different contexts. Its ambivalent behavior in tumors, in particular, has prompted in-depth investigations into its specific mechanisms. In NSCLC and CRC, NELL2 plays an oncogenic role through the E2F1 pathway, whereas in gastric, renal and osteosarcoma, it inhibits tumor progression through epigenetic silencing or regulation of the immune microenvironment. The role of NELL2 in tumorigenesis is complex and context-dependent, particularly with regard to its actions and signaling regulation within diverse TME.

Furthermore, the role of NELL2 in neurodegenerative diseases is still a matter of debate. For example, in AD, NELL2 overexpression can encourage the progression of pathology by inducing neuronal atrophy and increasing neuronal excitability. However, the balance between NELL2’s neuroprotective effects and its potential neurotoxicity is unclear. Its involvement in neurodevelopment, such as the regulation of axon guidance and neuronal differentiation, highlights its complexity in neurological function and disease.

NELL2 also plays a significant role in the immune system. It has been found to be closely associated with the function and infiltration of immune cells, and to act as an inflammatory regulator in immune-mediated diseases such as atopic dermatitis. However, the potential of NELL2 as a therapeutic target in different immune environments requires further validation.

However, this complexity also presents challenges and opportunities for precision interventions targeting NELL2. In recent years, as our understanding of the NELL2 regulatory network and signaling pathways has deepened, multiple targeting strategies have been proposed. These strategies encompass small-molecule inhibitors, non-coding RNAs, hormone regulation, splicing variants, antibody/protein therapeutics, and gene editing tools (**Table 1**). These approaches provide research tools for exploring the functional mechanisms of NELL2 and open new pathways for its clinical application in the treatment of cancer, neurodegenerative diseases, reproductive disorders and inflammatory diseases.

**Table 1:** Strategies in targeting NELL2.

Type of targeting	Concrete strategy	Mechanism of action
small molecule inhibitor	Genistein	Inhibits EGF signaling pathway and reduces NELL2 expression
	ACEE	Restoration of B[a]P-induced NELL2 downregulation
	EZH2 inhibitors(EPZ005687 GSK343)	Blockade of the NELL2-EZH2 axis activates the antiviral response and interferon pathway
non-coding RNA	miR-22 mimics	Inhibits NELL2 translation and suppresses tumor proliferation
	antisense oligonucleotide-targeted tRF-3017	Restoration of NELL2 expression and inhibition of gastric cancer metastasis
hormone regulation	E2	Activates NELL2 transcription and exerts neuroprotective effects by binding to the half ERE element of the NELL2 promoter via ERα/β
intracellular splice variants	cNELL2	Competitively binds to the pseudo-substrate domain of PKCβ1 and inhibits PKCβ membrane translocation and downstream ERK phosphorylation
antibody/protein therapy	anti-NELL2 neutralizing antibody	Blocking NELL2 binding to ROBO3 inhibits tumor growth
	recombinant NELL2 protein	NELL2 supplementation for neuronal survival and axonal regeneration
Gene editing tool	CRISPR knockout of NELL2	CRISPR mediated knockout of NELL2

Given the critical roles of NELL2 in the nervous system, development and disease, future studies could focus on elucidation of its molecular basis in various diseases, such as how the TME determines its oncogenic or oncostatic functions and its dynamic interactions with signaling molecules such as PKC and Robo3. To explore the potential of NELL2 as a prognostic marker (e.g. CRC, ESCC) or therapeutic target in cancer, e.g., by inhibiting the NELL2-EZH2 axis to activate the immune response in “cold tumors” should also be emphasized. The cross-species and evolutionary studies on NELL2 reveal its evolutionary significance in reproductive traits such as sperm maturation and follicular development, providing new ideas for animal breeding and human infertility treatment. In conclusion, as a cross-cutting molecular hub, NELL2 needs to be explored not only by deepening the basic mechanism, but also by developing novel therapeutic strategies through targeting NELL2.

## 8 Conclusion

As a neuron-specific secreted glycoprotein, NELL2 exhibits diverse roles in physiological and pathological processes such as reproductive development, immunomodulation, cancer, and neurodegenerative diseases. In the field of oncology, NELL2 exhibits a remarkable “dual role” as both an oncogene and a tumor suppressor. In the nervous system, NELL2 is involved in synaptic plasticity, axon guidance and hippocampal-dependent learning, and has been implicated in neuronal hyperexcitability and neuroprotective mechanisms in AD. In addition, in reproduction, NELL2 regulates sperm maturation through the lumicrine signaling pathway and its absence leads to male infertility. In the immune system, NELL2 is involved in the pathological processes of psoriasis through the regulation of Th2-type inflammatory responses and serves as a prognostic marker in TBM. Importantly, the molecular mechanisms of NELL2 in tissue-specific signaling interactions, the spatio-temporal dynamics of epigenetic regulation and the feasibility of therapeutic translation remain to be explored in depth.

## Glossary

AD: Alzheimer’s disease
ADs: Atopic dermatitis
AGO: Argonaute
ALT: Alternative lengthening of telomeres
BRP: Neuropathic pruritus
BTB: Blood-testis barrier
CC: Coiled-coil
CNPG: Chronic nodular pruritic dermatitis
CNS: Central nervous system
DCC: Deleted in colorectal cancer
DRG: Dorsal root ganglion
E2F1: E2F transcription factor 1
EMT: Epithelial-mesenchymal transition
ENK: Enkephalins
ER: Endoplasmic reticulum
ERVs: Endogenous retroviruses
ERα: E2 receptor alpha
ESCC: Esophageal squamous cell carcinoma
EZH2: Enhancer of Zeste Homolog 2
FN: Fibronectin type I
FTO: Fat mass and obesity-associated protein
GnRH: Gonadotropin-releasing hormone
HCC: Hepatocellular carcinoma
hiPSC: human induced pluripotent stem cell
LTP: Long-term potentiation
Macf1: Microtubule-actin crosslinking factor 1
MPNSTs: Malignant peripheral nerve sheath tumors
NEL: Neuro epidermal growth factor-like molecule
NELL: NEL-like
NELL2: Neural epidermal growth factor-like like 2
NICOL: NELL2-interacting cofactor for lumicrine signaling
NK: Natural killer
NSCLC: Non-small cell lung cancer
PKC: Protein kinase C
POA: Preoptic Area
PPE: Proenkephalinogen
PS: Pseudo-substrate
RARE: RA response elements
RCC: Renal cell carcinoma
RGC: Retinal ganglion cell
RISC: RNA-induced silencing complex
Robo: Roundabout
SDN-POA: Sexually dimorphic nucleus of the preoptic area
SP1REs: SP1 response elements
TBM: Tuberculous meningitis
TIL: Tumor-infiltrating lymphocyte
TME: Tumor microenvironment
TNBC: Triple-negative breast cancer
Tregs: Regulatory T cells
TSP-1: Thrombospondin-1
VDR: Vitamin D receptor
vWC: von Willebrand factor type C
WS: White striping
